# Retrospective Molecular Detection and Characterization of Pathogenic *Leptospira* in the Philippines

**DOI:** 10.3390/tropicalmed11030069

**Published:** 2026-03-04

**Authors:** Joanna Ina G. Manalo, Adeliza Mae L. Realingo, Lei Lanna M. Dancel, Timothy John R. Dizon, Amalea Dulcene Nicolasora, Kristine Alvarado-Dela Cruz, Desiree D. Argana, Arjay Niño A. Digman, Emarld Julian G. Medina, Celine Bernice A. Roxas, Rubelia A. Baterna, Julieta Z. Dungca

**Affiliations:** 1 Advanced Molecular Technologies Laboratory (AMTL), Research Institute for Tropical Medicine (RITM), Muntinlupa 1781, Philippines; adelizarealingo@gmail.com (A.M.L.R.);; 2 Graduate School, Centro Escolar University, Manila 1008, Philippines; 3 Department of Microbiology, Research Institute for Tropical Medicine (RITM), Muntinlupa 1781, Philippines; 4 School of Science and Technology, Centro Escolar University, Manila 1008, Philippines

**Keywords:** human leptospirosis, molecular diagnostics, 16s rRNA, Sanger sequencing, Philippine *Leptospira* infections

## Abstract

Leptospirosis remains a public health concern in the Philippines. Conventional diagnostic methods, including the microscopic agglutination test (MAT) and qPCR, are routinely used for outbreak response and surveillance. However, these methods often yield discordant results due to cross-reactivity, limited sensitivity, or lack of species-level resolution. To address these diagnostic gaps, this study optimized the Boonsilp 16S rRNA PCR assay and applied Sanger sequencing for accurate species identification of *Leptospira* in 92 archived DNA samples collected between 2018 and 2020. The sensitivity and specificity of the optimized assay were compared with those of MAT and qPCR. Species-level identification was confirmed via sequencing, and a phylogenetic tree was constructed. Among the 92 samples, 46 (50.0%) tested positive by qPCR, 39 (42.4%) by MAT, and 67 (72.8%) by at least one of the two methods. The optimized Boonsilp assay detected *Leptospira* in 23 samples (25.0%), of which 22 were also qPCR positive. Twenty-one samples were confirmed as *L. interrogans*, one as *L. borgpetersenii*, and one as an unclassified *Leptospira* species. One sample undetected by both MAT and qPCR tested positive using the optimized assay. Compared to the composite reference, the Boonsilp assay showed 32.8% sensitivity and 96.0% specificity. Phylogenetic analysis revealed multiple *L. interrogans* strains, including those closely related to reference sequences of Copenhageni, Manilae, and Canicola. While the optimized Boonsilp PCR assay demonstrates diagnostic value as an adjunct molecular tool to qPCR and MAT supporting species-level identification during outbreak surveillance, this warrants further validation in freshly isolated DNA samples.

## 1. Introduction

Leptospirosis is a global zoonotic disease caused by the flexible, spiral-shaped, Gram-negative spirochete bacteria known as *Leptospira* [1,2,3]. Among its many species, *Leptospira interrogans* is the most pathogenic, comprising over 300 serovars that infect both humans and animals [1,4,5]. It typically spreads to humans through contact with infected animal urine or contaminated environments [1,5]. Transmission typically occurs through direct or indirect contact with urine from infected animals, particularly in environments contaminated by floodwaters or animal waste [1]. Though initially classified as an occupational disease, leptospirosis is now recognized as a broader public health concern, especially in tropical and subtropical regions where outbreaks often follow heavy rainfall and flooding [2,6,7]. This ongoing burden emphasizes the need for sensitive, reliable tests to enhance case identification, especially during leptospirosis outbreak seasons.

In the Philippines, rats and large ruminants such as water buffalo and cattle have been identified as important reservoirs of pathogenic *Leptospira*, contributing significantly to human infections [6,8]. In animals, infection can lead to acute kidney dysfunction and, in some cases, fatal outcomes, often exacerbated by the host’s immune response [2,9,10]. Strains LT 398 (*L. interrogans* serovar Manilae), LT 101-69 (*L. interrogans* serovar Los Baños), and C3 (*L. interrogans* serovar Carlos) are among the well-characterized *Leptospira* isolates from the Philippines [11]. In Manila and Laguna (more precisely, Los Baños), rats yielded the first two isolates, and a toad yielded the third. A thorough grasp of Leptospirosis’ national status, specifically about prevalence, incidence, reservoirs, transmission channels, and virulence, is still lacking, even though numerous studies have looked at the disease in the Philippines. Notably, Mendoza and Rivera (2019) noted a steady rise in leptospirosis prevalence in urban settings, highlighting the significance of enhanced surveillance and molecular characterization for public health [12].

Serological testing remains integral to leptospirosis diagnosis, with the microscopic agglutination test (MAT) widely used to detect serovar-specific antibodies [13]. This method utilizes panel strains allowing detection of antibodies against specific *Leptospira* serovars [14]. Moreover, MAT necessitates bacterial culture in appropriately selected culture medium (Ellinghausen–McCullough–Johnson–Harris (EMJH)) [15,16]. The comprehensive culture requirements, along with the long incubation growth requirement of 10–14 days, highlight the need for more sensitive and specific tests to avoid misdiagnosing probable leptospirosis cases [2,10,17]. Additionally, MAT has reduced sensitivity in the early phase of infection, when antibody levels may still be undetectable, and it requires the labor-intensive maintenance of live *Leptospira* cultures for antigen preparation [17,18]. Seroconversion can take more than a week, leading to delays in diagnosis and treatment [19,20]. Despite these limitations, MAT remains the reference standard for serological diagnosis, though it cannot reliably differentiate between current, recent, or past infections. Its operational drawbacks, such as high cost, technical complexity, and biosafety concerns, make it less feasible for routine diagnostics, particularly in resource-limited settings [14,17,21,22].

At the Research Institute for Tropical Medicine (RITM), the *Leptospira* diagnostic workflow at the National Reference Laboratory for Leptospirosis (NRL-Leptospirosis) level comprises culture, MAT, and real-time PCR, which targets the *secY* gene of the pathogenic *Leptospira* species. Conventional PCR assays targeting the 16S rRNA gene, particularly the Boonsilp assay, offer an alternative approach [23]. When followed by Sanger sequencing, this method enables species-level identification and improved diagnostic resolution [23,24]. The increased sensitivity of 16S rRNA-targeted PCR is due to the fact that multiple copies of this gene are present in most bacterial genomes, unlike many single-copy target genes [23,25].

This study aimed to perform a comparative analysis of the positive and negative agreement of MAT, real-time PCR (qPCR targeting *secY*), and an optimized conventional PCR based on the Boonsilp assay targeting the 16S rRNA gene for the detection of *Leptospira* species using archived DNA samples collected in 2018–2020. We sought to identify diagnostic gaps and generate evidence that may lead to future improvements to the leptospirosis diagnostic testing algorithm and strengthen ongoing surveillance efforts. In this context, PCR amplification followed by sequencing of the 16S rRNA gene was also applied as a confirmatory approach to support species-level identification of *Leptospira* in samples that had tested positive by either MAT or qPCR.

## 2. Materials and Methods

### 2.1. Ethical Clearance

Ethical approval for this study was obtained from the RITM Institutional Review Board (RITM-IRB; IRB No. 2024-17) and the Centro Escolar University Institutional and Ethics Review Board (CEU-IERB; Protocol Code: CEU-IERB_2023–2024_317_GS). All patient metadata were anonymized, and only laboratory-assigned sample codes were used as identifiers to ensure confidentiality.

### 2.2. Study Design, Setting, and Sample Inclusion Parameters

This retrospective pilot study explored the use of the Boonsilp PCR assay, an optimized conventional PCR targeting the *Leptospira* 16S *rRNA* gene on archived DNA samples collected during the Philippine leptospirosis outbreaks from 2018 to 2020. These samples were selected based on availability, relevance to recent outbreaks, and the presence of both serological and molecular testing data. While the samples had been stored at −40 °C, potential DNA degradation over time could have impacted assay sensitivity and contributed to false-negative results. To better evaluate diagnostic performance, future studies should incorporate parallel testing using freshly collected samples alongside MAT and qPCR.

The study initially aimed to include 102 samples to achieve regional and temporal representation, targeting one sample per year from each of the 17 administrative regions of the Philippines over a three-year period (2018–2020), and to include both MAT-positive and MAT-negative cases. Due to budget constraints, the study design was refined to select one *Leptospira*-positive and one *Leptospira*-negative sample per region. Ultimately, 92 samples were obtained due to limitations in archived sample availability and quality. While this may not fully represent the diversity of *Leptospira* strains in each region, it offers a meaningful starting point to explore the assay’s utility across geographic and diagnostic contexts. As a proof-of-concept and pilot study, the findings are intended to guide future work with more comprehensive and systematically selected sample sets.

Samples were primarily selected based on qPCR results for pathogenic *Leptospira* (*secY* gene), prioritizing those with Cq values below 30 to capture higher bacterial loads, while also including intermediate and higher Cq values to reflect a range of bacterial concentrations. Selection followed predefined inclusion and exclusion criteria, with final selection based on sample availability, DNA quality, residual volume (≥100 µL), and appropriate storage conditions at or below −40 °C. All DNA extracts were processed at the Advanced Molecular Technologies Laboratory (AMTL), RITM, where they underwent Boonsilp PCR amplification and Sanger sequencing to assess the assay’s applicability for molecular detection. All Boonsilp PCR and Sanger sequencing procedures were conducted in 2024 using the archived DNA samples collected from 2018 to 2020.

### 2.3. Previously Performed Diagnostic Testing: MAT and qPCR Results

At the time of sample receipt, routine diagnostic testing was conducted by the NRL-Leptospirosis to support national surveillance and outbreak response. Samples underwent molecular detection using a qPCR assay targeting the *secY* gene specific to pathogenic *Leptospira* spp., following the method described by Ahmed et al. (2009) [26]. Positive results were based on amplification curves with Cq values ≤ 38. In parallel, the microscopic agglutination test (MAT) was performed using a panel of live *Leptospira* antigens maintained at RITM to identify circulating serovars. Positive results from either test were used to support case confirmation and surveillance reporting. Following diagnostic processing, DNA extracts were stored at −40 °C.

The MAT antigen panel used from 2018 to 2022 included commonly encountered reference serovars in the Philippines, although full details of the panel composition were not always available at the time of writing. The MAT reference standard was performed using live *Leptospira* strains maintained at the RITM NRL-Leptospirosis. Further details, including the specific serovars included in the MAT panel at the time of testing, are available in the Supplementary Material (Table S2).

For the present study, archived DNA samples were retrieved and re-tested in 2024 using an optimized 16S rRNA PCR assay to assess species-level identification and evaluate the performance of this alternative molecular method.

### 2.4. Molecular Detection Using Optimized PCR

An optimized Boonsilp PCR assay was used for the identification of *Leptospira* species. Positive and negative controls were tested in parallel with the samples to verify the validity of the PCR results. The positive control was anticipated to exhibit a band with a molecular weight of around 547 base pairs (bp) for the Boonsilp et al. (2011) [23] primers, while the negative control should not present any bands. Diluted nucleic acid of *L. interrogans* from an isolated culture provided by the RITM-Microbiology Department was used as a positive control for the optimization of the 16S rRNA PCR and sequencing assay following the Boonsilp (2011) protocol [23].

A touchdown PCR protocol was employed to enhance the Boonsilp assay for molecular detection, using the Q5^®^ Hot Start High-Fidelity 2X Master Mix (Catalog No. M0494L, New England Biolabs, Ipswich, MA, USA) and a C1000 PCR Thermal Cycler (Bio-Rad, Hercules, CA, USA). Each 25 µL reaction contained 12.5 µL of 2X master mix, 2.5 µL of DNA template, 1.25 pmol of each primer (rrs-inner-F: 5′-CTGGCGGCGCGTCTTA-3′ and rrs-inner-R: 5′-GTTTTCACACCTGACTTACA-3′), and nuclease-free water to reach the final volume. PCR cycling conditions began with an initial denaturation at 98 °C for 30 s. This was followed by 10 touchdown cycles consisting of denaturation at 98 °C for 10 s, annealing at 67 °C for 10 s (decreasing by 1 °C per cycle), and extension at 72 °C for 30 s. Subsequently, 35 additional cycles were performed with denaturation at 98 °C for 10 s, annealing at 59 °C for 15 s, and extension at 72 °C for 30 s. A final extension was carried out at 72 °C for 30 s. The expected amplicon size was 547 base pairs. PCR products were resolved on a 2% agarose gel stained with GelRed^®^ Nucleic Acid Gel Stain (Catalog No. B-41003, Biotium, Fremont, CA, USA) and visualized under UV transillumination to confirm amplicon size and specificity.

Positive PCR-amplified products were purified using QIAquick PCR Purification Kit (Qiagen, Hilden, Germany), and products were identified and verified by Sanger sequencing. Purified amplicons and the matching primers were used to construct sequencing reactions, which were then processed using the 3500 Genetic Analyzer Sanger sequencer (Applied Biosystems, Thermo Fisher Scientific, Waltham, MA, USA) based on capillary electrophoresis.

### 2.5. Phylogenetic Analysis

Sequences were compiled and modified after manual inspection of chromatogram quality. Consensus sequences were generated using MEGA version 11 (Molecular Evolutionary Genetics Analysis) [27], which incorporates the MUSCLE algorithm [28] for multiple sequence alignment. Closest *Leptospira* species matches were identified using BLASTn (accessed through the MEGA application and the NCBI web portal, https://blast.ncbi.nlm.nih.gov/Blast.cgi, in 30 July 2025; version available at the time of analysis), accessed through the MEGA application, and aligned with entries in the NCBI GenBank database.

To assess genetic relatedness, a phylogenetic tree was constructed based on the aligned consensus sequences. A maximum likelihood tree was generated using IQ-TREE version 2.2.0.5 [29], which automatically selected the best-fit substitution model. Bootstrap analysis (1000 replicates) was used to assess branch support. The resulting tree was visualized and annotated using FigTree version 1.4.4 [30].

This analysis contextualized the genetic similarities between local isolates and known reference strains, supporting species-level classification and revealing potential diversity among the detected *Leptospira* sequences.

### 2.6. Statistical Analysis

A statistical analysis was performed to evaluate the performance of the optimized Boonsilp assay using results from MAT and qPCR. Two sets of reference comparisons were made: (1) a composite reference standard consisting of samples positive by either MAT and/or qPCR, and (2) an individual comparison between qPCR and the Boonsilp assay.

Given the absence of a single definitive gold standard for *Leptospira* detection, we adopted a composite reference standard approach, combining results from both qPCR and MAT. This is in line with the rationale used in studies such as Dittrich et al. (2018) [31], where multiple diagnostic methods were considered together to overcome the limitations of individual assays. While their composite included culture, our approach relied on the combined strengths of qPCR and MAT to provide a more balanced estimation of diagnostic performance, recognizing the inherent limitations of each method.

For each comparison, 2 × 2 contingency tables were constructed. Sensitivity, specificity, and corresponding 95% confidence intervals were calculated where applicable. In other cases, especially where no true gold standard was available, positive and negative agreement rates were reported. A chi-square test of independence was applied to determine statistical significance, with a significance threshold of *p* < 0.05.

Statistical calculations were performed using Microsoft Excel and validated using online tools, including the MedCalc Diagnostic Test Calculator (MedCalc Software Ltd., version 23.2.6, https://www.medcalc.org/calc/diagnostic_test.php accessed 30 July 2025), DATAtab Chi-Square Test Calculator (DATAtab, https://datatab.net/statistics-calculator/hypothesis-test/chi-square_test_calculator; accessed 30 July 2025), and the Standard Deviation Chi-Square Calculator (https://www.standarddeviationcalculator.io/chi-square-calculator, accessed 30 July 2025). Frequencies and percentages were used to describe the distribution of identified *Leptospira* species in the samples. Cohen’s kappa statistic, along with its standard error and 95% confidence interval, was calculated using the MedCalc tool to evaluate agreement between the optimized Boonsilp PCR assay and the qPCR assay targeting pathogenic *Leptospira*.

This study adheres to the STARD 2015 guidelines for reporting diagnostic accuracy studies (see Supplementary File: STARD Checklist).

## 3. Results

**Figure 1:**
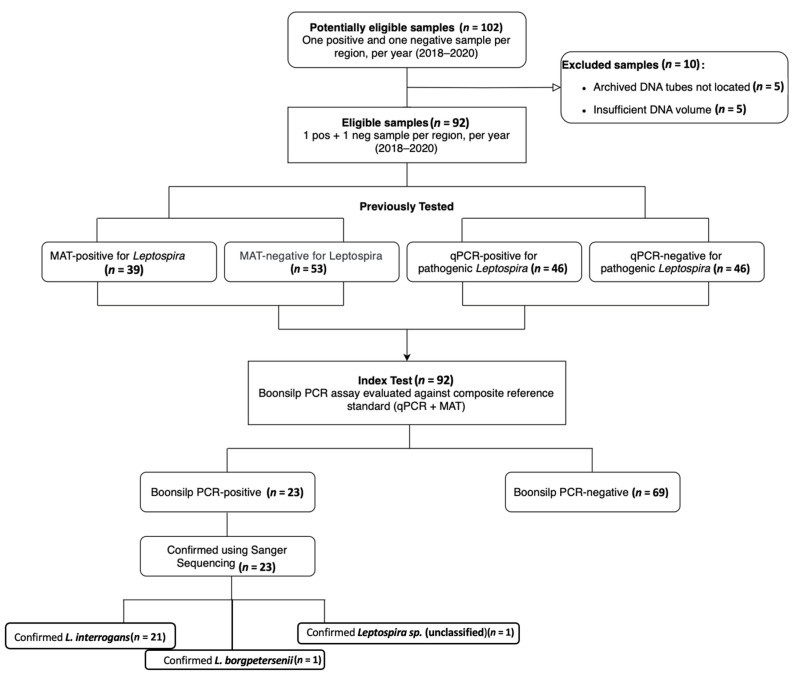
STARD flow diagram of diagnostic outcomes, testing, and sample selection. Eligibility was determined from 102 stored DNA samples. Ten samples were excluded while ninety-two underwent Boonsilp PCR testing and were assessed against a composite reference standard (qPCR + MAT). For species-level identification, PCR-positive samples were sequenced.

This study adapted and optimized the traditional 16S optimized Boonsilp PCR method developed by Boonsilp et al. (2011) [23], tailoring it for use with available laboratory resources and Philippine samples. The optimized assay enabled more accurate detection of *Leptospira* in both cultured isolates and clinical specimens. The STARD flow diagram (Figure 1) illustrates the inclusion process, the number of samples tested by each diagnostic method, and the corresponding positivity rates and overlaps among the assays.

### 3.1. PCR Optimization

For the optimization of the original Boonsilp assay [23], which uses outer and inner forward and reverse primers in a single tube amplification, the initial experiments following the original assay showed a primer dimer bands of about 50–100 bp. Upon primer analysis using Multiple Primer Analyzer [ThermoFisher Scientific, Waltham, MA, USA], it was noted that the outer primers exhibited self-complementarity, which led to the decision of omitting the primers from the mastermix. Primer dimers were successfully eliminated by using only the inner primers and optimizing their concentrations to enhance specificity and amplification efficiency.

### 3.2. Testing of Archived DNA Samples

**Figure 2:**
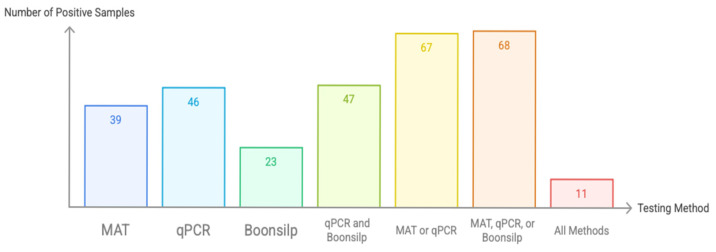
Number of confirmed leptospirosis patients based on MAT, qPCR, and optimized Boonsilp assay (*n* = 92).

Of the 92 archived DNA samples processed, 39 (42.4%) tested positive by MAT, 46 (50.0%) by qPCR, and 23 (25.0%) by the optimized Boonsilp PCR assay (Figure 2). When results of qPCR and optimized Boonsilp PCR assay were compared, it showed 47 (51.1%) overlap, while when using a composite reference standard (positive by either qPCR or MAT), 67 samples (72.8%) were considered positive. Notably, combining the results of all three assays would add only one additional sample to the composite reference standard. In total, only 11 samples (11.9%) tested positive across all three methods.

### 3.3. Diagnostic Performance of the Optimized Boonsilp Assay

Since no definitive gold standard exists for *Leptospira* diagnosis, this study used a composite reference standard, defined as a positive result from either MAT or qPCR, to evaluate the diagnostic performance of the optimized Boonsilp PCR assay. Table 1 presents the overall comparison, reporting a sensitivity of 32.84% (95% CI: 21.59–44.08%) and a specificity of 96% (95% CI: 88.32–100%).

To evaluate the diagnostic performance of the optimized Boonsilp PCR assay against a composite reference standard, results were compared using a 2 × 2 contingency table (Table 2). This table served as the basis for computing the diagnostic sensitivity and specificity reported above. The chi-square test yielded a statistic of 6.61 and a *p*-value of 0.0101, indicating a statistically significant difference at α = 0.05.

To further evaluate molecular concordance, the optimized Boonsilp assay was compared directly with qPCR, which targets the *secY* gene specific to pathogenic *Leptospira*. Among the 46 qPCR-positive samples, 22 (47.8%) were also positive by the Boonsilp assay. The calculated sensitivity was 46.81% (95% CI: 32.11–61.07%), while specificity reached 97.78% (95% CI: 93.47–100%) (Table 3). These results indicate that while the Boonsilp assay may have a lower detection rate compared to qPCR, it maintains high specificity and is effective for confirming the presence of *Leptospira* species, particularly when paired with sequencing.

A 2 × 2 contingency table (Table 4) was constructed to compare the results of the optimized Boonsilp PCR assay and the qPCR targeting pathogenic *Leptospira*. This table served as the basis for calculating sensitivity and specificity, as well as Cohen’s kappa statistic to assess agreement beyond chance. The observed agreement between the two assays was 71.7%. Cohen’s kappa statistic was 0.44 (95% CI: 0.29–0.60), indicating moderate agreement between the two molecular assays.

**Figure 3:**
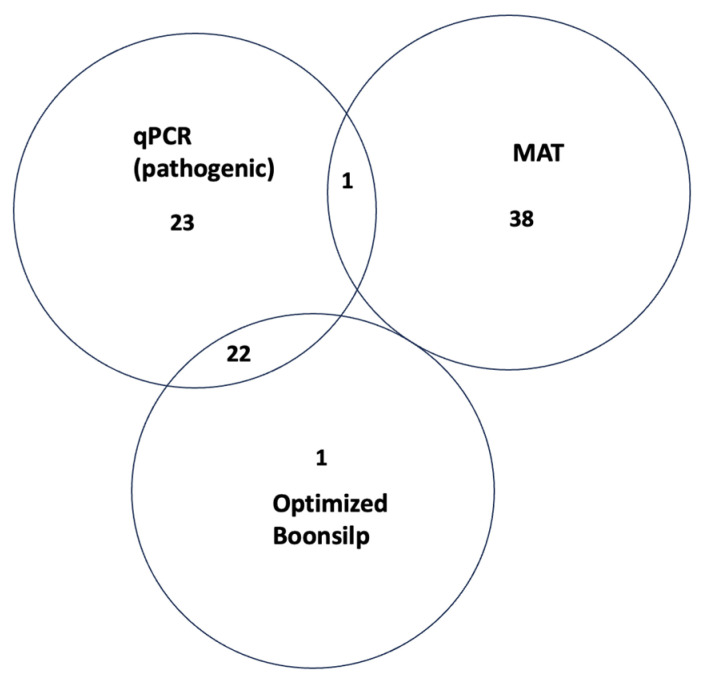
Venn diagram showing how many samples tested positive by MAT, qPCR for pathogenic *Leptospira*, and the optimized Boonsilp PCR assay. The overlap shows how the tests agree or differ in detecting *Leptospira* in the same samples.

Figure 3 illustrates the degree of concordance between the three diagnostic assays. A total of 22 samples tested positive by both qPCR and the optimized Boonsilp PCR assay. These samples were later confirmed by Sanger sequencing as either *L. interrogans* or *L. borgpetersenii*. One additional sample was also positive by both molecular assays and clustered with an unclassified *Leptospira* species based on sequencing results.

Only one sample (LS24-032) tested positive by both MAT and qPCR. Seventeen samples that tested positive by both MAT and qPCR did not yield positive results in the optimized Boonsilp assay and were previously classified by MAT as saprophytic *Leptospira* species. Fifteen samples were positive by MAT but negative by both molecular methods. Six samples were MAT-positive but negative by both qPCR and the optimized Boonsilp assay and were classified by MAT as pathogenic species.

One unique case (LS24-066) tested positive only by the optimized Boonsilp assay. Sanger sequencing confirmed the presence of *L. interrogans* in this sample, despite it testing negative by both MAT and qPCR.

The 23 Boonsilp-positive sequences were subjected to multiple sequence alignment and phylogenetic analysis, as described in Section 2.5. The resulting tree highlights the genetic diversity of *Leptospira* detected in the study population. The majority of sequences clustered within the *L. interrogans*, while one sequence grouped with *L. borgpetersenii*, and another aligned with an unclassified *Leptospira* species. Several *L. interrogans* sequences showed close similarity to GenBank reference sequences associated with serovars Copenhageni, Manilae, or Canicola. It should be noted, however, that due to the conserved nature of the 16S rRNA gene, these results do not permit definitive serovar-level identification and should be interpreted as species-level matches only. The species distribution of these Boonsilp-positive samples is summarized in Table 5.

**Figure 4:**
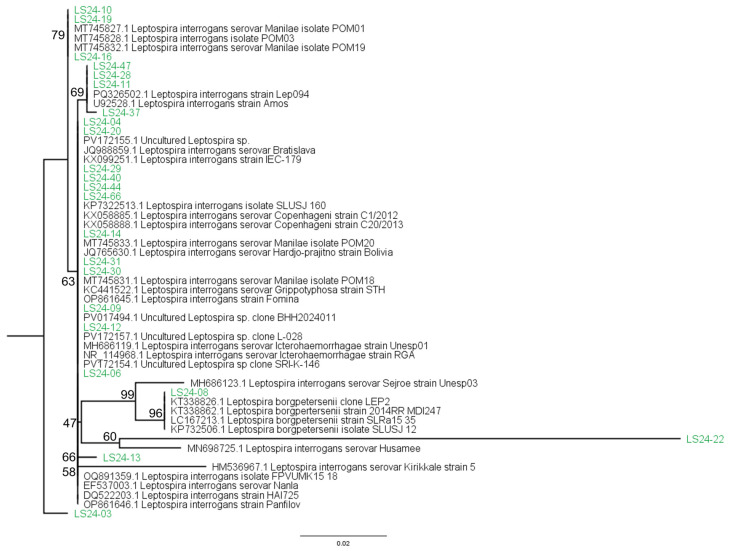
The phylogenetic tree illustrates the relationships between *Leptospira* partial sequences (black) obtained from the NCBI database and *Leptospira* sequences derived from human infections (green) in the Philippines, based on samples collected between 2018 and 2020.

The 16S rRNA sequences obtained in this study were submitted individually to GenBank using the NCBI sequence submission system, and accession numbers will be provided upon publication. The resultant phylogenetic tree (Figure 4) illustrates the relationships between sequenced samples and known *Leptospira* reference strains, providing preliminary insights into the species distribution of *Leptospira* in the Philippines.

### 3.4. Species Identification and Sequence Homology

BLAST analysis of the 16S rRNA amplicons yielded percent identities ranging from 98.81% to 100%, with query coverage between 98% and 100%, indicating robust sequence alignment. Most Boonsilp-positive samples showed high sequence homology to *Leptospira interrogans* reference sequences, suggesting that *L. interrogans* was the predominant species detected. Among these, several sequences aligned closely with GenBank entries associated with strains identified as serovars Copenhageni (e.g., CP048830.1), Manilae (e.g., CP011934.1), and Canicola (e.g., CP044513.1). While these alignments suggest similarity to commonly referenced strains, the conserved nature of the 16S rRNA gene does not permit definitive serovar-level identification.

In addition, one sample (LS24-047) showed close similarity to an uncultured *Leptospira* sequence (e.g., DQ887979.1), and another sample (LS24-048) matched *Leptospira borgpetersenii*. These findings may reflect the presence of underrepresented or uncharacterized *Leptospira* strains in the study population. One additional sample, (LS24-022) aligned with *L. interrogans* in the phylogenetic tree and blast search, but its sequence could not be successfully uploaded to NCBI due to repeated submission errors. Although it was classified as *L. interrogans* based on phylogenetic analysis, further testing may be warranted if remaining sample volume permits. Alternatively, they may highlight the limited taxonomic resolution of the 16S rRNA gene, which can make it challenging to distinguish closely related species. All alignments had E-values of 0, indicating high-confidence matches.

## 4. Discussion

To the best of our knowledge, this is the first study in the Philippines to evaluate an optimized molecular assay, adapted from Boonsilp et al., as a complementary tool for *Leptospira* detection using archived human DNA samples [23]. This assay was assessed in parallel with the current diagnostic methods employed by the NRL-Leptospirosis, namely quantitative PCR (qPCR) targeting pathogenic species and the microscopic agglutination test (MAT) [26]. While both methods are widely used, they present notable limitations: qPCR lacks species-level resolution, and MAT is susceptible to cross-reactivity, variable sensitivity, and delayed antibody responses, particularly in early infection stages [14,23,32,33].

Introducing a molecular method that not only detects *Leptospira* but also allows for species-level identification offers valuable enhancements in diagnostic precision and public health surveillance [23,34]. The optimized Boonsilp PCR assay, targeting the 16S rRNA (*rrs*) gene, paired with Sanger sequencing, enabled the identification of multiple *Leptospira* species, including *L. interrogans*, *L. borgpetersenii*, and an unclassified strain. This capability is especially critical in endemic regions like the Philippines, where molecular epidemiological data remain limited and MAT panels may not reflect circulating strains [24,35].

Although the Boonsilp assay yielded the lowest detection rate among the three methods (25.0%), this can likely be attributed to DNA degradation in archived samples, which may have reduced amplification efficiency [32,34,35]. Nevertheless, its value becomes evident when considering concordance and confirmatory capability. A key finding was the high agreement between qPCR and the optimized Boonsilp assay: 22 samples were positive by both methods and confirmed as pathogenic via sequencing. Additionally, one sample (LS24-066) tested positive only by the Boonsilp assay and was confirmed as *L. interrogans*, suggesting that this assay may offer broader strain coverage or improved sensitivity beyond the reach of qPCR primers and MAT antigens [23,24]. However, this finding is based on a single case and does not provide sufficient evidence to establish diagnostic superiority; further validation through additional testing and larger sample sets is necessary.

Although MAT detected 42.4% of samples as positive, its overlap with molecular methods was limited, raising concerns about specificity. Only one MAT-positive sample (LS24-032) also tested positive by qPCR and represented a true pathogenic infection. Interestingly, 17 samples tested positive in both MAT and qPCR. However, while qPCR targets only pathogenic *Leptospira*, the MAT results for these samples indicated saprophytic species, raising questions about possible cross-reactivity or misclassification by MAT. This discrepancy suggests possible cross-reactivity in MAT or detection of antibodies from non-pathogenic exposures rather than active infection [23,33]. Additionally, 15 samples were MAT-positive but qPCR-negative, with MAT results which may indicate non-pathogenic *Leptospira* species considering that qPCR is specific for pathogenic *Leptospira* species [36]. It is interesting to note that six samples were reported to be positive in pathogenic *Leptospira* in MAT, but surprisingly yielded negative results by both qPCR and the optimized Boonsilp assay, which may suggest false-positive results [32,33]. Similar to our findings, Podgoršek et al. (2020) reported discordant results between RT-PCR and conventional PCR targeting the 16S rRNA gene, attributing some discrepancies to DNA degradation in archived samples. In their study, a conventionally PCR-positive but RT-PCR–negative sample had been stored for four years, raising concerns about nucleic acid integrity in retrospective testing [37]. As with their study, we also used archived DNA samples, which may have contributed to instances where qPCR or MAT results did not align with Boonsilp PCR outcomes. We acknowledge that sample degradation could have affected assay performance, particularly for low-copy targets, and recommend follow-up testing using freshly extracted DNA from prospectively collected clinical samples to further validate the assay’s diagnostic value.

While MAT detects an immune response, which can persist long after infection or arise from non-pathogenic exposure, molecular methods detect *Leptospira* DNA during active infection [9,23,32,33,35]. This difference in timing between what each test detects helps explain some of the mismatched results. PCR is most effective during the early, bacteremic phase when the bacteria are still present in the blood, while MAT detects antibodies that usually appear later in the illness [14,33,34,38]. This highlights the value of PCR-based methods for identifying infections in their early stages [26,35]. A limitation of this study is the lack of detailed MAT serovar data and titers, which may have contextualized discrepancies between molecular and serological methods.

The phylogenetic analysis of Boonsilp-positive samples further confirmed the assay’s utility. Most were clustered under *L. interrogans* (Copenhageni, Manilae, Canicola), with one case of *L. borgpetersenii*, and another aligning with an unclassified *Leptospira* species. These data not only validate the assay’s specificity but also highlight the presence of strains not well represented in current reference databases, reinforcing the importance of sequencing in surveillance. Despite showing lower sensitivity when compared to a composite reference standard, the optimized Boonsilp assay demonstrated high specificity and offered distinct diagnostic value through species-level confirmation, as 16S rRNA PCR reliably differentiates pathogenic from non-pathogenic *Leptospira* [33,39]. Its modest sensitivity partly reflects the use of an imperfect reference (culture + MAT), which undervalues molecular methods [14,22]. When compared solely to qPCR, the Boonsilp assay showed stronger concordance, highlighting its value in strengthening diagnostic confidence and enabling species-level resolution where standard tools may fall short [23,26,40].

While 16S rRNA sequencing helped confirm *Leptospira* species in our samples, it does not provide enough detail to identify specific serovars or closely related strains. In our dataset, some sequences aligned with reference strains labeled as Copenhageni, Manilae, or Canicola, but these should be understood as species-level matches only. Because the 16S gene is highly conserved, it is not suitable for fine-scale typing. For molecular epidemiology or outbreak tracking, more variable targets like *secY*, *lipL32*, or MLST are recommended instead [24,39]. Our findings highlight the importance of interpreting 16S-based data cautiously, particularly in the context of public health surveillance, where misclassification may impact outbreak tracking and response.

MAT interpretation depends heavily on expertise and is prone to variability, especially when using live cultures and panels that may not represent all circulating strains [17,41,42]. Differences in sample types, archived DNA for the Boonsilp assay versus fresher DNA for MAT and qPCR validation, may have also impacted detection rates. Several studies have raised concerns about MAT’s cross-reactivity and variable specificity, especially in endemic areas where individuals are frequently exposed to environmental leptospires [9,14,22]. A recent animal study by Mummah et al. (2024) also found that host species can influence MAT results due to varying immune responses [42]. Similarly, Denipitiya et al. (2016) noted substantial discordance between MAT and qPCR, with MAT-positive but PCR-negative results possibly reflecting later-stage samples or residual antibodies [32].

Moreover, while MAT and culture remain historical gold standards [9,10,14,43], their low sensitivity and operational limitations make them less ideal for routine clinical diagnostics. Culture is slow and rarely positive after antibiotic administration, and MAT’s complexity makes it impractical for use outside reference laboratories [14,33,41]. Hence, molecular methods, particularly PCR targeting multicopy genes like *rrs*, are increasingly preferred for early, sensitive detection [10,26,32]. This study reinforces these observations.

Although the 16S rRNA gene is still a commonly utilized marker for identifying and detecting *Leptospira*, taxonomic resolution may be limited by its conserved nature [25,40,44]. Our phylogenetic tree showed that sample LS24-08, which was determined to be *L. borgpetersenii*, was closely associated with *L. interrogans*. Previous studies have identified similar limits and suggest that for better species-level categorization, more selective markers as *secY* or multilocus sequence typing should be used [24,26,45,46,47,48]. Future studies may benefit from incorporating MLST schemes to better resolve closely related strains and to complement single-gene phylogenetic approaches.

Targeting the 16S *rRNA* gene (*rrs*), which exists in two genomic copies, may enhance detection sensitivity, especially in low-DNA samples. Previous research [25,33,38,49] also supports this choice, showing better performance of *rrs*-based assays over *lipL32*-based assays in certain clinical scenarios. Finally, the optimized Boonsilp assay, though not intended to replace current tests, adds a valuable layer to diagnostic workflows. Its strength lies in enabling species-level identification and confirming cases that may otherwise be ambiguous, especially in regions where multiple species co-circulate or where MAT panel coverage is insufficient. The sequencing of Boonsilp-positive cases revealed underrepresented species and even unidentified strains, demonstrating the importance of incorporating genomic surveillance into leptospirosis diagnostics. In places like the Philippines, where species-level identification is not routinely performed, incorporating molecular tools such as the Boonsilp assay can aid in the surveillance efforts and guide more targeted public health interventions.

Ultimately, combining molecular and serological tools with sequencing enhances both diagnostic accuracy and pathogen monitoring [14,22]. However, it is important to acknowledge that the assays used in this study differ not only in target but also in diagnostic principle and analytical sensitivity [10,26,50]. Conventional PCR assays like the Boonsilp assay may have a higher limit of detection compared to qPCR, which has been shown in other studies [26] to detect lower concentrations of leptospiral DNA through real-time fluorescence monitoring [51]. Meanwhile, MAT captures host antibodies that may persist beyond the infectious period and may not reflect current bacterial load [52]. While formal LOD comparisons were not performed here, these fundamental differences likely contribute to assay discordance and highlights the challenges of comparing molecular and serological tests.

## 5. Conclusions

This pilot study highlights the potential diagnostic and epidemiological value of the optimized Boonsilp PCR as a complementary assay to MAT and qPCR. While its detection rate was relatively lower for archival DNA, its strength lies in species-level identification and its ability to resolve discordant results between molecular and serological methods. It is recommended that future studies using DNA isolated from fresh clinical samples be made to further validate its diagnostic performance and assess its practical utility. As such, the inclusion of the optimized Boonsilp PCR followed by Sanger sequencing to the diagnostic workflows can greatly improve early detection and identification of *Leptospira* species during outbreak response and surveillance efforts, thus supporting the WHO Global Genomic Surveillance Strategy.

## Figures and Tables

**Table 1:** Performance evaluation of the composite reference (positive for either MAT or qPCR) against the optimized Boonsilp assay for *Leptospira* detection.

Composite Reference Test vs. Optimized Boonsilp PCR Assay		95% CI
Sensitivity	32.84%	21.59% to 44.08%
Specificity	96%	88.32% to 100%

Note: Composite reference assay (positive for either MAT or qPCR).

**Table 2:** Contingency table comparing the composite reference (positive for either MAT or qPCR) against the optimized Boonsilp assay for *Leptospira* detection.

Composite Reference Assay	Boonsilp Positive	Boonsilp Negative	Total
*Leptospira*-tested positive	22	1	23
*Leptospira*-tested negative	45	24	69
Total	67	25	92

Note: Composite reference assay (positive for either MAT or qPCR).

**Table 3:** Performance evaluation of qPCR and the optimized Boonsilp assay for *Leptospira* detection.

*secY* qPCR Assay vs. Optimized Boonsilp PCR Assay		95% CI
Sensitivity	46.81%	32.11% to 61.07%
Specificity	97.78%	93.47% to 100%

**Table 4:** Contingency table comparing qPCR and optimized Boonsilp PCR assay results (*n* = 92).

qPCR Result	Boonsilp Positive	Boonsilp Negative	Total
qPCR Positive	22	1	23
qPCR Negative	25	44	69
Total	47	45	92

**Table 5:** Distribution of *Leptospira* species and sequence-level matches to reference strains using the optimized Boonsilp assay.

*Leptospira* Species/Serovars	Closest GenBank Reference Match *	Number of Positive Samples	Percent (%)
*L. interrogans* serovar Copenhageni	Copenhageni	14	60.87
*L. interrogans* serovar Manilae	Manilae	4	17.39
*L. interrogans* serovar Canicola	Canicola	3	13.04
*L. borgpetersenii*	-	1	4.35
*Leptospira* sp. (unclassified)	unclassified	1	4.35
Total		23	100

* Closest GenBank reference match is based on BLAST analysis of partial 16S rRNA sequences. These do not imply confirmed serovar-level identification.

## Data Availability

The data presented in this study are available on request from the corresponding author. GenBank accession numbers for all submitted sequences are included in the Supplementary Materials. Clinical metadata are not publicly available due to privacy and ethics restrictions.

## Supplementary Materials

The following supporting information can be downloaded at: https://www.mdpi.com/article/10.3390/tropicalmed11030069/s1, Table S1: Comparative Results of qPCR, MAT, and Optimized Boonsilp PCR Assay with Sequencing for 92 Archived DNA Samples (2018–2020); Table S2: Summary of *Leptospira* Serovars Detected by MAT (2018–2020); Table S3: GenBank Accession Numbers of *Leptospira* Sequences; STARD 2015 Checklist: Reporting guideline checklist for the study titled “Retrospective Molecular Detection and Characterization of Pathogenic *Leptospira* in the Philippines”.
