# Supplementary material for: Retrospective Molecular Detection and Characterization of Pathogenic Leptospira in the Philippines
Source: Trop Med Infect Dis. 2026 Mar 4;11(3):69. doi: 10.3390/tropicalmed11030069 (PMC13030237; doi:10.3390/tropicalmed11030069)
Supplement: Supplementary file 1 [file tropicalmed-11-00069-s001.zip › tropicalmed-3730227-supplementary.pdf]

Supplemental Materials

Supplementary Table S1. Comparative Results of qPCR, MAT, and Optimized Boonsilp PCR Assay with Sequencing for 92 Archived DNA Samples (2018–2020).

#	Unique Code	QPCR RESULT	MAT RESULT	Optimized Boonsilp Assay
1	LS24-001	Positive	Negative	Negative
2	LS24-003	Positive	Negative	*Leptospira interrogans* serovar Manilae strain
3	LS24-004	Positive	Negative	*Leptospira interrogans* serovar Copenhageni strain
4	LS24-005	Positive	Negative	Negative
5	LS24-006	Positive	Negative	*Leptospira interrogans* serovar Copenhageni strain
6	LS24-007	Positive	Negative	Negative
7	LS24-008	Positive	*L. biflexa* serovar Patoc	*Leptospira borgpetersenii* strain
8	LS24-009	Positive	*L. biflexa* serovar Patoc	*Leptospira interrogans* serovar Copenhageni strain
9	LS24-010	Positive	*L. biflexa* serovar Patoc	*Leptospira interrogans* serovar Manilae strain
10	LS24-011	Positive	*L. fainei* serovar Hurstbridge	*Leptopira interrogans* serovar Canicola strain
11	LS24-012	Positive	*L. biflexa* serovar Patoc	*Leptospira interrogans* serovar Copenhageni strain
12	LS24-013	Positive	Negative	*Leptospira interrogans* serovar Copenhageni strain
13	LS24-014	Positive	Negative	*Leptospira interrogans* serovar Copenhageni strain
14	LS24-015	Positive	Negative	Negative
15	LS24-016	Positive	Negative	*Leptospira interrogans* serovar Manilae strain
16	LS24-017	Positive	*L. biflexa* serovar Patoc	Negative
17	LS24-018	Positive	Negative	Negative
18	LS24-019	Positive	Negative	*Leptospira interrogans* serovar Manilae strain
19	LS24-020	Positive	Negative	*Leptospira interrogans* serovar Copenhageni strain
20	LS24-021	Positive	Negative	Negative
21	LS24-022	Positive	*L. biflexa* serovar Patoc	*Leptospira interrogans* serovar Copenhageni strain
22	LS24-023	Positive	*L. biflexa* serovar Patoc	Negative
23	LS24-024	Positive	*L. biflexa* serovar Patoc	Negative
24	LS24-025	Positive	Negative	Negative
25	LS24-026	Positive	*L. biflexa* serovar Patoc	Negative

26	LS24-027	Positive	Negative	Negative
27	LS24-028	Positive	*L. biflexa* serovar Patoc	*Leptospira interrogans* Canicola strain
28	LS24-029	Positive	*L. fainei* serovar Hurstbridge<br>*L. biflexa* serovar Patoc	*Leptospira interrogans* serovar Copenhageni strain
29	LS24-030	Positive	*L. biflexa* serovar Patoc	*Leptospira interrogans* serovar Copenhageni strain
30	LS24-031	Positive	*L. fainei* serovar Hurstbridge<br>*L. biflexa* serovar Patoc	*Leptospira interrogans* serovar Copenhageni strain
31	LS24-032	Positive	*L. interrogans* serovar Canicola<br>*L. fainei* serovar Hurstbridge<br>*L. biflexa* serovar Patoc	Negative
32	LS24-033	Positive	*L. biflexa* serovar Patoc	Negative
33	LS24-034	Positive	Negative	Negative
34	LS24-035	Positive	Negative	Negative
35	LS24-036	Positive	Negative	Negative
36	LS24-037	Positive	Negative	*Leptospira interrogans* Canicola strain
37	LS24-038	Positive	Negative	Negative
38	LS24-039	Positive	Negative	Negative
39	LS24-040	Positive	Negative	*Leptospira interrogans serovar Copenhageni strain*
40	LS24-041	Positive	*L. biflexa* serovar Patoc	Negative
41	LS24-042	Positive	Negative	Negative
42	LS24-043	Positive	Negative	Negative
43	LS24-044	Positive	*L. biflexa* serovar Patoc	*Leptospira interrogans* serovar Copenhageni strain
44	LS24-045	Positive	Negative	Negative
45	LS24-046	Positive	Negative	Negative
46	LS24-047	Positive	Negative	*Leptospira* sp.
47	LS24-048	Negative	Negative	Negative
48	LS24-049	Negative	Negative	Negative
49	LS24-050	Negative	Negative	Negative
50	LS24-051	Negative	*L. biflexa* serovar Patoc	Negative
51	LS24-052	Negative	*L. fainei* serovar Hurstbridge	Negative
52	LS24-053	Negative	Negative	Negative

53	LS24-054	Negative	*L. borgpetersenii* serovar Javanica	Negative
54	LS24-055	Negative	*L. interrogans* serovar Carlos<br>*L. biflexa* serovar Patoc	Negative
55	LS24-056	Negative	*L. biflexa* serovar Patoc	Negative
56	LS24-057	Negative	Negative	Negative
57	LS24-058	Negative	*L. biflexa* serovar Patoc	Negative
58	LS24-059	Negative	*L. fainei* serovar Hurstbridge	Negative
59	LS24-060	Negative	*L. fainei* serovar Hurstbridge<br>*L. biflexa* serovar Patoc	Negative
60	LS24-061	Negative	Negative	Negative
61	LS24-062	Negative	*L. biflexa* serovar Patoc	Negative
62	LS24-063	Negative	Negative	Negative
63	LS24-064	Negative	Negative	Negative
64	LS24-065	Negative	*L. biflexa* serovar Patoc	Negative
65	LS24-066	Negative	Negative	*Leptospira interrogans* serovar Copenhageni strain
66	LS24-067	Negative	Negative	Negative
67	LS24-068	Negative	Negative	Negative
68	LS24-069	Negative	Negative	Negative
69	LS24-070	Negative	*L. biflexa* serovar Patoc	Negative
70	LS24-071	Negative	*L. interrogans* serovar Autumnalis<br>*L. biflexa* serovar Patoc	Negative
71	LS24-072	Negative	Negative	Negative
72	LS24-073	Negative	Negative	Negative
73	LS24-074	Negative	*L. biflexa* serovar Patoc	Negative
74	LS24-075	Negative	*L. interrogans* serovar Carlos	Negative
75	LS24-078	Negative	Negative	Negative
76	LS24-079	Negative	Negative	Negative
77	LS24-080	Negative	*L. biflexa* serovar Patoc	Negative
78	LS24-081	Negative	*L. biflexa* serovar Patoc	Negative
79	LS24-082	Negative	Negative	Negative
80	LS24-083	Negative	Negative	Negative

81	LS24-084	Negative	*L. fainei* serovar Hurstbridge<br>*L. biflexa* serovar Patoc	Negative
82	LS24-085	Negative	Negative	Negative
83	LS24-086	Negative	*L. biflexa* serovar Patoc	Negative
84	LS24-087	Negative	Negative	Negative
85	LS24-088	Negative	*L. interrogans* serovar Bataviae<br>*L. fainei* serovar Hurstbridge<br>*L. biflexa* serovar Patoc	Negative
86	LS24-090	Negative	*L. interrogans* serovar Canicola<br>*L. interrogans* serovar Icterohaemorrhagiae	Negative
87	LS24-092	Negative	Negative	Negative
88	LS24-093	Negative	*L. fainei* serovar Hurstbridge<br>*L. biflexa* serovar Patoc	Negative
89	LS24-094	Negative	Negative	Negative
90	LS24-095	Negative	Negative	Negative
91	LS24-096	Negative	Negative	Negative
92	LS24-097	Negative	Negative	Negative

Supplementary Table S2. Summary of *Leptospira* Serovars Detected by MAT (2018–2020).

Year	Serovar	Number of Positive Samples
2018	Autumnalis	16
	Bataviae	12
	Carlos	13
	Icterohaemorrhagiae	4
	Javanica	1
	Losbanos	1
	Pomona	6
	Hurstbridge	57
	Patoc	343
2019	Canicola	69
	Carlos	6

	Icterohaemorrhagiae	1
	Javanica	3
	Pomona	1
	Hurstbridge	128
	Patoc	400
2020	Icterohaemorrhagiae	1
	Javanica	7
	Losbanos	1
	Pomona	5
	Hurstbridge	84
	Patoc	98

Supplementary Table S3. GenBank Accession Numbers of *Leptospira* Sequences.

Sample ID	Accession Number
Leptospira/Philippines/LS24-03	PV998119
Leptospira/Philippines/LS24-04	PV998120
Leptospira/Philippines/LS24-06	PV998121
Leptospira/Philippines/LS24-08	PV998122
Leptospira/Philippines/LS24-09	PV998123
Leptospira/Philippines/LS24-10	PV998124
Leptospira/Philippines/LS24-11	PV998125
Leptospira/Philippines/LS24-12	PV998126
Leptospira/Philippines/LS24-13	PV998127
Leptospira/Philippines/LS24-14	PV998128
Leptospira/Philippines/LS24-16	PV998129
Leptospira/Philippines/LS24-19	PV998130
Leptospira/Philippines/LS24-20	PV998131
Leptospira/Philippines/LS24-28	PV998132
Leptospira/Philippines/LS24-29	PV998133
Leptospira/Philippines/LS24-30	PV998134
Leptospira/Philippines/LS24-31	PV998135
Leptospira/Philippines/LS24-37	PV998136
Leptospira/Philippines/LS24-40	PV998137
Leptospira/Philippines/LS24-44	PV998138
Leptospira/Philippines/LS24-47	PV998139
Leptospira/Philippines/LS24-66	PV998140

Supplemental Materials

STARD 2015 Checklist for Retrospective Molecular Detection and Characterization of Pathogenic *Leptospira* in the Philippines

Item No.	STARD Checklist Item	Reported on Page/Section
1	Identification as a study of diagnostic accuracy	Title page, Abstract
2	Structured summary of study design, methods, results, and conclusions	Abstract
**INTRODUCTION**		
3	Scientific and clinical background, including the intended use and clinical role of the index test	Introduction
4	Study objectives and hypotheses	Introduction, last paragraph
**METHODS**		
5	Whether data collection was planned before the index test and reference standard were performed (prospective study) or after (retrospective study)	Methods 2.2 ("retrospective pilot study")
**Participants**		
6	Eligibility criteria for participants	Methods 2.2 (archived DNA from suspected leptospirosis cases with available MAT/qPCR data)
7	On what basis potentially eligible participants were identified (e.g., symptoms, results from previous tests)	*Methods 2.2 (qPCR-positive or MAT-available samples from outbreak testing at RITM)*
8	Where and when potentially eligible participants were identified (setting, location, dates)	Methods 2.2 (Philippines, samples from 2018–2020)
9	Whether participants formed a consecutive, random or convenience series	*Methods 2.2 – The study aimed for regional and temporal representation, selecting one MAT-positive and one MAT-negative sample per region per year, based on available archived DNA with sufficient volume and quality.*
**Test methods**		
10a	Index test, in sufficient detail to allow replication	Methods 2.4
10b	Reference standard, in sufficient detail to allow replication	Methods 2.3
11	Rationale for choosing the reference standard	*Methods 2.3 + Discussion (composite standard due to absence of gold standard)*

12a	Definition of and rationale for test positivity cut-offs for index test	Methods 2.4 (PCR positive = amplification of 547 bp product + sequencing confirmation)
12b	Definition of and rationale for test positivity cut-offs for reference standard	Methods 2.3 (qPCR Ct < 30 prioritized; MAT titers not available acknowledged as limitation)
13a	Whether clinical information and reference standard results were available to the performers/readers of the index test	*Not blinded. PCR and sequencing were performed by researchers who may have had access to previous MAT and qPCR results, as full blinding was not feasible during assay optimization and sequencing analysis.*
13b	Whether clinical information and index test results were available to assessors of the reference standard	*Not applicable, MAT and qPCR were done prior to this study*
**Analysis**		
14	Methods for estimating diagnostic accuracy measures	Methods 2.5
15	How indeterminate index test or reference standard results were handled	*Methods 2.2 (No indeterminate results; add note: "Only samples with valid MAT and qPCR results were included.")*
16	How missing data on the index test and reference standard were handled	*Methods 2.2 ("Ten samples were excluded due to insufficient DNA volume or degraded quality.")*
17	Analyses of variability in diagnostic accuracy	*Not applicable (no subgroup analysis performed)*
18	Intended sample size and how it was determined	Methods 2.2 (Targeted 102 for regional/yearly representation; final n = 92 due to constraints)
**RESULTS**		
**Participants**		
19	Flow of participants, using a diagram	Figure 1 (STARD diagram)
20	Baseline demographic and clinical characteristics	*Not available; retrospective archived DNA only (stated in Discussion as a limitation)*
21a	Distribution of severity of disease in those with the target condition	*Not available, acknowledged as a limitation.*
21b	Distribution of alternative diagnoses in those without the target condition	*Not available, acknowledged as a limitation.*
**Test results**		

22	Time interval and any clinical interventions between index test and reference standard	*Methods 2.2 and Discussion (Index testing done in 2024; on archived DNA from 2018–2020)*
23	Cross-tabulation of the index test results by the results of the reference standard	Results Table 5
24	Estimates of diagnostic accuracy and their precision	Results Tables 3 and 4 (with 95% CI)
25	Any adverse events from performing the index test or reference standard	Not applicable (no patient contact; archived samples only)
**DISCUSSION**		
26	Study limitations, including sources of potential bias, statistical uncertainty, and generalizability	Discussion (includes retrospective design, missing MAT details, no clinical data)
27	Implications for practice, including the intended use and clinical role of the index test	Discussion and Conclusion (Boonsilp as complementary/species-level tool)
**OTHER INFORMATION**		
28	Registration number and name of registry	Not applicable – retrospective study
29	Where the full study protocol can be accessed	*No protocol registered; not publicly available*
30	Sources of funding and role of funders	Acknowledgments – No external funding; laboratory resources provided by RITM; funders had no role in study design, analysis, or manuscript preparation.
